# High-quality genome resource of *Lasiodiplodia pseudotheobromae* associated with die-back on *Eucalyptus* trees

**DOI:** 10.1186/s12863-023-01187-6

**Published:** 2024-01-02

**Authors:** LinQin Lu, GuoQing Li, FeiFei Liu

**Affiliations:** Research Institute of Fast-growing Trees (RIFT), Chinese Academy of Forestry (CAF), Zhanjiang, 524022 China

**Keywords:** Reference genome, Comparative genome, Function annotation, Fungal disease, Canker

## Abstract

**Objectives:**

*Lasiodiplodia pseudotheobromae* is an important fungal pathogen associated with die-back, canker and shoot blight in many plant hosts with a wide geographic distribution. The aim of our study was to provide high-quality genome assemblies and sequence annotation resources of *L. pseudotheobromae*, to facilitate future studies on the systematics, population genetics and genomics of the fungal pathogen *L. pseudotheobromae.*

**Data description:**

High-quality genomes of five *L. pseudotheobromae* isolates were sequenced based on Oxford Nanopore technology (ONT) and Illumina HiSeq sequencing platform. The total size of each assembly ranged from 43 Mb to 43.86 Mb and over 11,000 protein-coding genes were predicted from each genome. The proteins of predicted genes were annotated using multiple public databases, among the annotated protein-coding genes, more than 4,300 genes were predicted as potential virulence genes by the Pathogen Host Interactions (PHI) database. Moreover, the genome comparative analysis among *L. pseudotheobromae* and other closely related species revealed that 7,408 gene clusters were shared among them and 152 gene clusters unique to *L. pseudotheobromae*. This genome and associated datasets provided here will serve as a useful resource for further analyses of this fungal pathogen species.

## Objective

Members of *Botryosphaeriaceae* are considered as latent pathogens and can infect numerous hosts almost all woody plants [1]. Diseases associated with them usually occur under environmental stresses such as drought, frost and heat, and typical symptoms include canker, dieback, root rot, fruit rot and twig blight [1, 2]. *Lasiodiplodia pseudotheobromae* (*Botryosphaeriaceae*, *Botryosphaeriales*) was first described in 2008, which is closely related to *L. theobromae* [3]. The known hosts include nearly 100 species in 40 families, such as forest trees of *Eucalyptus* spp., *Acacia* spp., *Pinus* spp., crop plants of *Gossypium hirsutum*, *Citrus* spp., and ornamental plants of *Bougainvillea spectabilis*, *Magnolia candolei* [4]. The geographic distribution of this pathogen recorded includes China [5–7], Malaysia [8], Brazil [9], Venezuela [10], South Africa [11], Tunisia [12] and Spain [13].

In southern China, studies on *Botryosphaeriaceae* showed that *L. pseudotheobromae* is one of the dominant causal agents of *Eucalyptus* die-back, canker and shoot blight in plantations, especially in [6, 14]. Inoculation trials in the greenhouse and field suggested that this pathogen has a relatively high virulence to different *Eucalyptus* species or hybrids, compared to other species in *Botryosphaeria* and *Neofusicoccum* [7]. For this important pathogen, there are three isolates with publicly available genomic data in the NCBI database, CBS 116459 from *Gemlina arborea* [15], KET9 from *Prunus persica* [16] and BaA from *Morinda officinalis* [17] (DataFile 1; Table 1) [18]. These genome assemblies are fragmented and not suitable as reference genomes. Thus, high-quality genome assemblies based on long-read sequencing technology by Oxford Nanopore Technologies (ONT) were conducted in this study. These new genomic resources can provide more information for future studies aimed at fungal biology and pathogenic mechanism of *L. pseudotheobromae*.

**Table 1:** Overview of data files/data sets

Label	Name of data file/data set	File types (file extension)	Data repository and identifier (DOI or accession number)
Data file 1	Table S1, Genome assembly and annotation features of *Lasiodiplodia pseudotheobromae* isolates	Document file (.docx)	Figshare, 10.6084/m9.figshare.24419041.v3 [18]
Data file 2	Figure S1, Venn diagram	Image file (.jpg)	Figshare, 10.6084/m9.figshare.24418999.v1 [19]
Data file 3	Figure S2, Phylogenetics analyses	Image file (.jpg)	Figshare, 10.6084/m9.figshare.24419029.v1 [20]
Data set 1	Genome assembly of *Lasiodiplodia pseudotheobromae* strain RIFT 3495	Fasta file (.fasta)	NCBI Nucleotide, https://identifiers.org/nucleotide: JAWMWM000000000 [21]
Data set 2	Genome assembly of *Lasiodiplodia pseudotheobromae* strain RIFT 6050	Fasta file (.fasta)	NCBI Nucleotide, https://identifiers.org/nucleotide: JAWMWL000000000 [22]
Data set 3	Genome assembly of *Lasiodiplodia pseudotheobromae* strain RIFT 15092	Fasta file (.fasta)	NCBI Nucleotide, https://identifiers.org/nucleotide: JAWMWK000000000 [23]
Data set 4	Genome assembly of *Lasiodiplodia pseudotheobromae* strain RIFT 18431	Fasta file (.fasta)	NCBI Nucleotide, https://identifiers.org/nucleotide: JAWMWJ000000000 [24]
Data set 5	Genome assembly of *Lasiodiplodia pseudotheobromae* strain RIFT 19273	Fasta file (.fasta)	NCBI Nucleotide, https://identifiers.org/nucleotide: JAWMWI000000000 [25]

## Data description

Five *L. pseudotheobromae* isolates originated from plantation trees of *Eucalyptus* spp. and *Cunninghamia lanceolata* in southern China were selected for genome sequencing in this study (DataFile 1; Table 1) [18]. Fresh mycelia of the single hyphal tip isolates were harvested from 2% MEA plates (20 g malt extract powder and 20 g agar per litre of water) covered with cellophane for 2 days at 25 °C and immediately frozen in liquid nitrogen, followed by preservation at -80 °C in the laboratory prior to DNA extraction. High-quality genomic DNA was extracted using a modified CTAB (cetyltrimethylammonium bromide) method [26]. The integrity and purity of DNA were detected by 0.8% agarose gel electrophoresis and the precise concentration of which was quantified by a Qubit 2.0 fluorescence detector (Life Technologies). All five isolates were confirmed as *L. pseudotheobromae* by sequencing the elongation factor 1-α (*EF1-α*) gene and phylogenetic analyses.

Whole genome sequencing was conducted using both the short-read platform and the long-read Oxford Nanopore Technologies (ONT) in Zhenyue Biotechnology Co., Ltd (WuHan, China). The Illumina sequencing was performed for all the five isolates (RIFT3495, RIFT 6050, RIFT 15092, RIFT 18431 and RIFT 19273). Paired-end library with 350 bp median insert size was generated and 150 bp paired-end reads were sequenced using the Illumina HiSeq 2500 platform. Poor-quality data and adapters were removed using the program Trimmomatic v. 0.36 [27]. The program SPAdes v. 3.14 [28] was used to assemble the genome *de novo* into contigs. The ONT sequencing was performed for the two isolates RIFT 3495 and RIFT 18431. The library was loaded on a MinION R10.3 flow cell (FLO-MIN111) and the sequencing run was carried out for 48 h. Base calling was conducted using the ONT Guppy base calling software v. 4.0.14 (https://community.nanoporetech.com). GenomeScope was used to estimate the size of genomes [29]. The ONT reads were assembled with the program Mecat2 (20,190,226) with default parameters after filtration of the low-quality reads [30]. The assembled genome was then polished with ONT reads and Illumina reads by using Racon v. 1.4.11 [31] and Pilon v. 1.23 [32], respectively.

Genome size of the five strains were generated by GenomeScope, ranging from 42 to 44.61 Mb, and the heterozygosity was estimated to be 0.01 to 0.24%. An average of 2,081,811 ONT reads (up to 332 × coverage) and 49,479,273 Illumina clean reads (up to 192 × coverage) were generated in this study (DataFile 1; Table 1) [18]. The assembled draft genomes were about 43 Mb in size and with the highest N_50_ value (5,817,267 bp) and the minimum contig numbers (8 contigs) among all the published *L. pseudotheobromae* genomes (DataFile 1; Table 1) [15–17, 33]. For each of the five genomes, a perfect spectra graph performed by KAT program [34] was acquired, clearly showing a complete haplotype achieved. Benchmarking Universal Single-Copy Orthologs (BUSCO) based on fungi_odb 10 [35] was used to evaluate the completeness of the genome assemblies. The results showed a high completeness score of up to 99.2% of all the five assemblies in this study, which indicated that the continuity of these assemblies is comparable with the publicly available genomes but is essentially better than them (DataFile 1; Table 1) [15–17, 33].

Maker2 v. 2.31.9 [36] was used for *de novo* gene prediction. In total, up to 12,237 genes were predicted as protein-coding genes with an average length of 1,937.92 bp for all the five genomes in this study (DataFile 1; Table 1) [18]. In addition, about 245 noncoding RNAs (transfer RNA, ribosomal RNA and small nuclear RNA) were predicted using tRNAscan-SE v. 2.0 [37] and Barmap v. 0.8 (https://github.com/tseemannbarmap). Further, repeat family identification and modeling were performed *de novo* using Repeatmasker v. 4.0.7 [38]. An average of 59,444 bp of repeat sequences that accounted for about 0.14% of the assemblies were detected in the assembled genomes (DataFile 1; Table 1) [18].

## Annotation

Functional annotation of the predicted gene sequences was done using BLAST to search against multiple public databases, including the lnterProScan database (ave. 8,453 genes, 73.76%), Gene Ontology (GO; ave. 1,858 genes, 16.21%), Kyoto Encyclopedia of Genes and Genomes (KEGG; ave. 10,868 genes, 94.82%), Swiss-Prot database (ave. 7,323 genes, 63.91%), TrEMBL database (ave. 11,410 genes, 99.62%) and NCBIs Nonredundant Protein (Nr; ave. 11,453 genes, 99.91%). Additional annotation was carried out based on the Pathogen Host Interactions (PHI) database [39], and Carbohydrate-Active Enzymes (CAZys) databases [40]. Meanwhile, secretory proteins were analyzed using Signal P v. 4.1 and TMHMM v. 2.0 [33]. A total of average 4,429 (PHI) genes were identified in the five genomes, and nearly 900 genes of each genome were annotated from the CAZys databases, including 405 genes related to glycoside hydrolases (GHs), 185 genes related to glycosyl transferases (GTs), 57 genes related to carbohydrate esterases (CEs), 28 genes related to polysaccharide lyases (PLs), 108 genes predicted to have auxiliary activities (AAs) and 87 genes associated with carbohydrate-binding modules (CBMs). Moreover, a total of average 835 putative secondary proteins were identified in the five genomes.

The comparative genomics of the orthologous gene cluster between *L. pseudotheobromae* RIFT 3495 and three related species (*Lasiodiplodia theobromae*, *Botryosphaeria dothidea*, *Neofusicoccum parvum*) were analyzed using the CD-HIT v. 4.6.1 rapid clustering of similar proteins software with a threshold of 50% pairwise identity and 0.7 length difference cutoff in amino acids, which revealed 7,408 common gene clusters and 152 gene clusters unique to RIFT 3495. RIFT 3495 shared 786, 93 and 13 gene clusters with *L. theobromae*, *B. dothidea* and *N. parvum*, respectively (DataFile 2; Table 1) [19]. Software RAxML was used to construct the evolutionary tree by the maximum likelihood method [41], phylogenetic analysis of single copy orthologous genes from twelve genomes along with *Aplosporella prunicola* (as outgroup) showed a similar association of *L. pseudotheobromae* with *L. theobromae*, followed by *Diplodia corticola* and *D. seriata* (DataFile 3; Table 1) [20].

This study presents five draft genome sequence resources of *L. pseudotheobromae*, a fungal pathogen causing trunk disease in southern China, which is of great importance for elucidating the biology and pathogenicity of this fungus on woody perennial trees.

## Limitation

The *de novo* assemblies resulted in a number of contigs, the genomic quality of the three *L. pseudotheobromae* isolates which sequenced only based on the Illumina Hiseq platform were still fragmented and not suitable for genome structure analysis. Further high-quality genome assemblies using long-read sequencing technologies for those isolates are still needed.

## Data Availability

The data described in this Data note were deposited under NCBI BioProject ID PRJNA1030934 [19–23]. Associated Datafiles are available on Figshare: Table S1, Genome assembly and annotation features of *Lasiodiplodia pseudotheobromae* isolates [18], Figure S1, Venn diagram [19], Figure S2, Phylogenetics analyses [20]. Please see Table 1 for details and links to the data.
